# Exact Weighted-FBP Algorithm for Three-Orthogonal-Circular Scanning Reconstruction

**DOI:** 10.3390/s90604606

**Published:** 2009-06-12

**Authors:** Hongli Hu, Jianzhou Zhang

**Affiliations:** College of Computer, Sichuan University, 610065, Chengdu, P. R. China

**Keywords:** cone-beam reconstruction, three-orthogonal scanning, filtering lines, weighting function

## Abstract

Recently, 3D image fusion reconstruction using a FDK algorithm along three-orthogonal circular isocentric orbits has been proposed. On the other hand, we know that 3D image reconstruction based on three-orthogonal circular isocentric orbits is sufficient in the sense of Tuy data sufficiency condition. Therefore the datum obtained from three-orthogonal circular isocentric orbits can derive an exact reconstruction algorithm. In this paper, an exact weighted-FBP algorithm with three-orthogonal circular isocentric orbits is derived by means of Katsevich's equations of filtering lines based on a circle trajectory and a modified weighted form of Tuy's reconstruction scheme.

## 1. Introduction

The cone-beam scanning configuration with a circular trajectory remains one of the most popular scanning configuration and has been widely employed for data acquisition in 3-D X-ray computed tomography (CT), because it allows for operating at a high rotating speed due to its symmetry, avoiding the need to axially translate the patient, such as in helical or step-and-shoot CT [1, 2]. Unfortunately, a circular trajectory does not satisfy Tuy's data sufficiency condition [3] which requires every plane that passes through the reconstruction region (ROI) must also cut the trajectory at least once and therefore only approximate reconstructions are possible. In the pursuit of the data sufficiency condition, various scanning trajectories have been proposed, such as circle and line [4, 5], circle plus arc [6, 7], double orthogonal circles [8, 9] and dual ellipses [10].

Recently, the FDK algorithm [11] has been implemented for a cone-beam vertex trajectory consisting of three-orthogonal isocentric circles [12]. On the other hand, we know that 3D image reconstruction based on three-orthogonal circular isocentric orbits is sufficient in the sense of Tuy data sufficiency condition. Therefore the datum obtained from three-orthogonal circular isocentric orbits can derive an exact reconstruction algorithm. In this paper, another theoretically exact and general inversion formula which was proposed in Katsevich [13] will be implemented for three-orthogonal circular isocentric orbits. Compared with the aforementioned algorithms, the distinctive features of Katsevich's algorithm can be summarized as the choice of a more general weight and a novel way to dealing with discontinuous in weight. Furthermore, Katsevich's algorithm has been implemented for various scanning trajectories, such as circle and line [14, 15], circle plus arc [16], several circular segment [17], two orthogonal circles [13] and helix [18, 19].

In contrast to a singular circular trajectory, there are several advantages in using weighted reconstruction with the three-orthogonal-circular trajectory. First, for the reconstruction noise due to the algorithm is ’trajectory’ dependent. Using three-orthogonal geometry with weighting function, the noise is suppressed. Second, the three-orthogonal setting yields better image quality in comparison with classical scheme for larger cone-angles. Last, the scan method for three-orthogonal geometry can be implemented with minimal requirements and can provide a better 3D reconstruction for small animals or objects. At the same time, compared to the two-circular trajectory, artifacts of the boundaries of the ROI where artifacts are likely to appear are suppressed by the additional filtering lines and according weighting functions.

In our implementation, a new weighting scheme is developed so that all measurements are used by accurately averaging over multiply measured projections of the three-orthogonal-circular scanning. Moreover, this new weighted reconstruction is differs from the weighted FDK [11] reconstruction with the three-orthogonal-circular trajectory [12] considerably, in that it is a theoretically exact formulation of a general shift invariant filtered back-projection (FBP) reconstruction framework. In this paper, we show the derivation of our cone-beam FBP reconstruction algorithm starting with Tuy's inversion formula. In other words, we discuss how to derive a FBP cone-beam reconstruction formula from the Tuy's classical inversion formula. To construct the reconstruction formula, several operators are needed. First, using some properties of the cone-beam transform, the classical Tuy's inversion formula can be written as (the first-order derivative of the Radon transform in ℝ^3^) Grangeat's inversion formula [20] in a weighted form. Then, using the methodology in[13], we can get the final inversion formula. The features of our inversion formula can be summarized as follow: using some properties of the cone-beam transform to derive the inversion formula, a proper choice of the weighting function, deriving equations of the filtering lines and describing geometric properties of filtering lines in the planar detector plane.

The organization of this paper is as follows. In Section 2, we derive Katsevich's inversion formulation by Tuy's weighted form. In Section 3, we derive equations of the filtering planes and filtering lines. In Section 4, we show the geometric properties of filtering lines in the planar detector plane and according weighting functions.

## 2. Weighting Function for Tuy's Formula

Let a trajectory *C* be a differentiable curve in ℝ^3^ described by *y*(*s*), *s* ∈ ℝ. The object density function is *f*(*x*), where *x* is a vector in the (*y*_1_, *y*_2_, *y*_3_) coordinate system, and *f*(*x*) is an infinitely differentiable real integrable function with a compact support Ω ⊂ ℝ^3^\*C*. The modified cone-beam projection of *f*(*x*) along the direction of *α*/‖*α*‖ at the focal point location *y*(*s*) is defined as:

(1) $$Df(y(s),\alpha )=\int_{0}^{\infty }f(y+t\alpha )dt=\frac{1}{\left||\alpha |\right|}Df(y(s),\frac{\alpha }{\left||\alpha |\right|}),(y(s),\alpha )\in C\times S^{2}.$$

Then, let *Df* be extended from *C* × 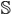
^2^ to ℝ^3^ × ℝ^3^ as:

(2) $$D_{y}f(z)=Df(y,z),y,z\in {\mathbb{R}}^{3}.$$

First consider the three-dimensional Fourier transform of *D_y_f*(*z*) for a fixed *f*(*x*) as:

(3) $$\tilde{D_{y}f}(\sigma )={\int_{{\mathbb{R}}^{3}}Df(y,z)e}^{-2\pi iz\cdot \sigma }dz.$$

It can be easily verified that:

(4) $$\begin{array}{cc} \tilde{D_{y}f}(t\sigma )=\frac{1}{t^{2}}\tilde{D_{y}f}(\sigma ),\;\text{for} & \;t>0. \end{array}$$

In the following, we will show how to derive a new reconstruction formula from Tuy's reconstruction scheme in a weighted form.

### Theorem 1

[Tuy's weighted form] Let *C* be a curve in ℝ^3^ parameterized by a piecewise differentiable function *y*(*s*), *s* ∈ ℝ. For a fixed *x* ∈ Ω, we suppose that there exists a weighting function *n_x_*(*s, σ*) : ℝ × 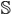
^2^ → ℝ such that *n_x_*(*s, σ*) is integrable with respect to the second variable *σ* ∈ 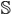
^2^ for each *s* ∈ ℝ, and the set:

(5) R(x,σ)={s∈ℝ|x⋅σ=y(s)⋅σ,y′(s)⋅σ≠0}

is non-empty and finite for almost all *σ* ∈ 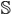
^2^. Then:

(6) $$f(x)=\int_{{\text{S}}^{2}}\sum_{s\in R(x,\sigma )}\frac{n_{x}(s,\sigma )}{2\pi iy^{\prime }(s)\cdot \sigma }\frac{\partial }{\partial q}\tilde{D_{y(q)}f}(\sigma ){|}_{q=s}d\sigma$$

provided that *n_x_*(*s, σ*) fulfilled the completeness condition:

(7) $$\sum_{s\in R(x,\sigma )}n_{x}(s,\sigma )=1,a.e.in\sigma \in {\text{S}}^{2}.$$

This inversion formula also tells us Tuy's data sufficiency conditions for an accurate reconstruction of a ROI from cone-beam projections. These conditions are: *σ* · *y*(*s*) = *σ* · *x* and *σ* · *y'*(*s*) ≠ 0. In the following, we will show how to derive a FBP reconstruction formula from Tuy's formula and show how Tuy's data sufficiency and nonended condition can be relaxed. In a first step we try to use Equation (1) to simplify Equation (4) as follows:

(8) $$\tilde{iD_{y}f}(\sigma )=i{\int_{R^{3}}Df(y(q),z)e}^{-2\pi iz\cdot \sigma }dz=i\int_{S^{2}}\int_{0}^{\infty }\frac{1}{r}Df(y(q),\theta )e^{-2\pi iz\cdot \sigma }r^{2}drd\theta ,$$

where *r* is the spherical radical coordinate, *θ* is a three dimensional unit vector such that *z* = *rθ* and ‖*θ*‖ = 1. Next we introduce the quantity:

(9) $$i\tilde{D_{y}f}(\sigma )=\frac{1}{2}\int_{S^{2}}Df(y(q),\theta )d\theta \int_{-\infty }^{\infty }ire^{-2\pi ir\theta \cdot \sigma }dr+\frac{1}{2}\int_{S^{2}}Df(y(q),\theta )d\theta \int_{-\infty }^{\infty }i|r|e^{-2\pi ir\theta \cdot \sigma }dr.$$

In Equation (7) the first term is real and add, and the second term is imaginary and even if we take into account the fact that the factor *y'*(*s)* · *σ* in the inversion formula is also odd in *σ*, we conclude that only the first term contributes in Tuy's inversion formula. The second term will vanish when we perform the integration over *σ.* Thus we have:

(10) $$i\tilde{D_{y}f}(\sigma )=\frac{-1}{4\pi }\int_{{\text{S}}^{2}}Df(y(q),\theta )\delta^{\prime }(\theta \cdot \sigma )d\theta .$$

Thus, we rederived the weighted form of Tuy's inversion formula as follows:

(11) $$f(x)=\frac{1}{8\pi^{2}}\int_{{\text{S}}^{2}}\sum_{s\in R(x,\sigma )}\frac{n_{x}(s,\sigma )}{y^{\prime }(s)\cdot \sigma }\int_{{\text{S}}^{2}}\frac{\partial }{\partial q}Df(y(q),\theta ){|}_{q=s}\delta^{\prime }(\theta \cdot \sigma )d\theta d\sigma .$$

Therefore, there is a derivative of delta function in the integration over *θ.* We can perform integration by part for the integration over *θ* in Equation (10). We thus obtain the directional derivative along the direction *σ* of cone-beam data $\tilde{Df}(y(q),\theta )$ with respect to unit vector *θ.* Therefore Equation (10) can be written as follows:

(12) $$f(x)=-\frac{1}{8\pi^{2}}\int_{{\text{S}}^{2}}\sum_{s\in R(x,\sigma )}\frac{n_{x}(s,\sigma )}{y^{\prime }(s)\cdot \sigma }\frac{\partial }{\partial q}\{\int_{{\text{S}}^{2}}\nabla_{\theta ,\sigma }Df(y(q),\theta )\delta (\theta \cdot \sigma )\}{|}_{q=\text{s}}d\theta d\sigma .$$

Using Grangeat's formula and change of variables *ρ* → *q* defined by *ρ* = *y*(*q*) · *σ,* we obtain:

(13) $$\frac{1}{y^{\prime }(s)\cdot \sigma }\frac{\partial }{\partial q}\left\{\int_{S^{2}}\nabla_{\theta ,\sigma }Df(y(q),\theta )d\theta \right\}{|}_{q=s}=\frac{\partial^{2}}{\partial \rho^{2}}\hat{f}(\rho ,\sigma ){|}_{\rho =y(s)\cdot \sigma =x\cdot \sigma },$$

where *f̂*(*ρ, σ*) is the 3D Radon transform of *f*. Modifications from Tuy's original inversion (6) to Equation(12) with change of variables have been important progress. Accounting for the numerical implementation of formula Equation (11) is still complicated. We can change the two integrals over the unit vector *θ* = (−sin *γ* cos *φ,* sin *γ* cos *φ,* cos *γ*) and *σ* = (cos *φ,* sin *φ,* 0) into three single integrals over parameters s, *φ* and *γ*. Using the methodology in [13], we get:

(14) $$f(x)=-\frac{1}{8\pi^{2}}\int_{I}\frac{ds}{\left|x-y(s)\right|}\int_{0}^{2\pi }\frac{\partial }{\partial \varphi }\{\text{sgn}(y^{\prime }(s)\cdot \sigma )n_{x}(s,\sigma )\}d\varphi \times \int_{0}^{2\pi }\frac{\partial }{\partial q}Df(y(q),\cos\gamma \beta (s,x)+\sin\gamma e_{x}(s,\varphi )){|}_{q=s}\frac{d\gamma }{\sin\gamma }$$

where *β*(*s, x*) = (*x* − *y*))/‖ *x* − *y*(*s*)‖ = (0,0,1),*e_x_*(*s, φ*) = *β*(*s, x*) × *σ_x_*(*s, φ*), denote

(15) $$\begin{array}{l} \kappa_{x}(s,\varphi )=\text{sgn}(y^{\prime }(s)\cdot \sigma )n_{x}(s,\varphi )\\ w_{m}(s,x)=\underset{ɛ\to 0}{\lim}(k_{x}(s,\varphi_{m}+ɛ)-k_{x}(s,\varphi_{m}-ɛ)). \end{array}$$

The values of *w_m_*(*s, x*) are determined by the definition of the weighting function and signum function near the discontinuous points *φ_m_*'s. Furthermore, in the FBP reconstruction scheme, the unit vector *σ_x_*(*s, φ_m_*) is the normal vector of filtering plane and the vector *e_x_*(*s, φ_m_*) denotes the direction of filtering lines which pass through the object point *x* and the source point *y(s)*. After substituting Equation (14) into formula Equation (13), we obtain:

(16) $$f(x):=-\frac{1}{8\pi^{2}}\int_{I}\sum_{m}\frac{w_{m}(s,x)}{\left|x-y(s)\right|}\times \int_{0}^{2\pi }\frac{\partial }{\partial q}Df(y(q),\cos r\beta (s,x)+\sin re_{x}(s,\varphi m)){|}_{q=s}\frac{dr}{\sin r}ds.$$

The final inversion formula is independent of the specific geometrical shape of the image object. Rather, it is determined by the scanning geometry Thus, we need study the specific features of filtering lines and weighting functions. In the following, the inversion formula will be implemented for a cone-beam vertex trajectory consisting of three-orthogonal to each other circular. Furthermore, the filtering lines and the weighting functions will be given in detail.

## 3. Equation of Filtering Lines for the Three-Orthogonal Circular Scanning

**Figure 1.**
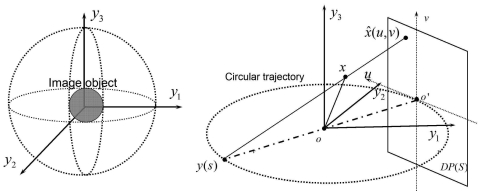
Geometries for three-orthogonal circular trajectory and the planar-detector plane.

We propose the use of three independent, orthogonal to each other, circular isocentic scan-paths for X-ray projection acquisitions. Let a trajectory *C* = *C*_1_ ∪ *C*_2_ ∪ *C*_3_, as shown in Figure 1.

where:

(17) $$C_{1}=\{y(s)\in R^{3}:y_{1}(s)=R\cos s,y_{2}(s)=R\sin s,y_{3}(s)=0,s\in [0,2\pi ]\};$$

(18) $$C_{2}=\{y(s)\in R^{3}:y_{1}(s)=R\cos s,y_{2}(s)=0,y_{3}(s)=R\sin s,s\in [2\pi ,4\pi ]\};$$

(19) $$C_{3}=\{y(s)\in R^{3}:y_{1}(s)=0,y_{2}(s)=R\cos s,y_{3}(s)=R\sin s,s\in [4\pi ,6\pi ]\}.$$

Let *f*(*x*) is the density function to be constructed. Assume that the function is smooth and vanishes outside the ball:

(20) $$\Omega =\{x=(y_{1},y_{2},y_{3})|y_{1}^{2}+y_{2}^{2}+y_{3}^{2}\leq r\},0<r<R,$$

where *r* is the radius of the subject ball and R is the radius of the scanning circular. We assume that the physical detector is a planar-detector, which is denoted as *DP*(*s*), where s is the parameter of source point. Furthermore, let the planar-detector is at distance 2*R* from the source, as shown in Figure 1. Denote by (*u, v*) the horizontal and vertical coordinates of the planar-detector plane, where *u* is parallel to the tangent vector of source, *v* is the normal vector of scanning trajectory and the origin is at the projection of the source point *y*(*s*) onto the detector plane.

Therefore, in the (*y*_1_, *y*_2_, *y*_3_) coordinate system, any point on the detector plane can be characterized completely by use of (*u, v*). It can be shown that:

(21) $$y_{1}=u\sin s-R\cos s,y_{2}=-u\cos s-R\sin s,y_{3}=v,\;s\in [0,2\pi ];$$

(22) $$y_{1}=u\sin s-R\cos s,y_{2}=v,y_{3}=-u\cos s-R\sin s,\;s\in (2\pi ,4\pi ];$$

(23) $$y_{1}=v,y_{2}=u\sin s-R\cos s,y_{3}=-u\cos s-R\sin s,\;s\in (4\pi ,6\pi ].$$

**Figure 2.**
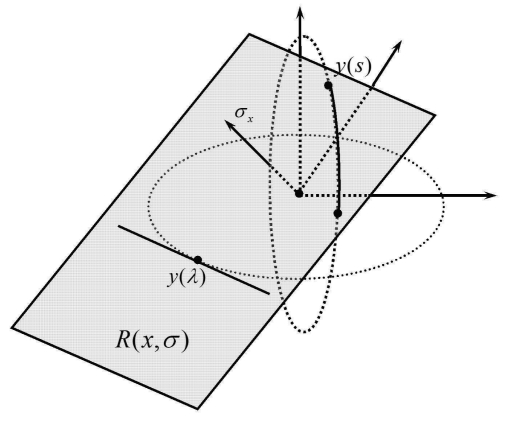
The filtering plane is shown, which is tangent to one of circular trajectories and is perpendicular to σx.

Figure 2 shows a filtering plane *R(x, σ)* through the *y(s)* and is tangent to other trajectory at point *y*(λ). Without loss of generality we consider the source *y(s)* ∈ *C*_1_ (the case *y(s)* ∈ *C*_2,3_ is treated in a similar fashion). The point of tangency is given by either *y*(λ) = (*R* cosλ, 0, *R* sinλ) or *y*(λ) =(0, *R* cosλ, *R* sinλ). In the first case, the normal vector to the tangent plane is given by:

(24) $$\sigma_{x}=(\sin s\cos\lambda ,1-\cos s\cos\lambda ,\sin s\sin\lambda ).$$

The equation of tangent plane, which is a filter plane, is given by:

(25) R(x,σ)={x|(x−y(s))⋅σ=0}.

Filtering lines on the detector are obtained by intersecting the detector plane and filtering planes. Substituting Equation (20) and Equation (23) into Equation (24) and solving for *v,* we obtain the equation of the filtering line on the planar-detector:

(26) $$v(u:s,\lambda )=\frac{u(\cos s-\cos\lambda )}{\sin s\sin\lambda }+\frac{2R}{\sin\lambda }.$$

In the second case, the normal vector to the tangent plane is given by:

(27) e=(1−cosscosλ,cosscosλ,cosssinλ).

The equation of the filtering line on the planar-detector is given by:

(28) $$v(u:s,\lambda )=\frac{u(\cos\lambda -\sin s)}{\sin\lambda \cos s}+\frac{2R}{\sin\lambda }.$$

## 4. Weighting Functions of Filtering Lines

**Figure 3.**
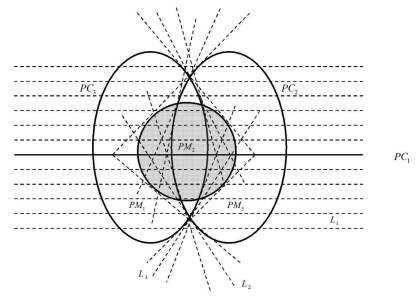
Detector plane with various filtering lines covering projection of ROI shown.

In the following, we define adequate filtering lines for the three-orthogonal scanning case. In order to define the different filtering lines, which are necessary to perform the three-orthogonal circular reconstruction, we first, introduce certain curves, which separate the planar-detector into different regions. As an example consider the X-ray source moving on the trajectory *C*_1_. We project stereographically the trajectories *C*_2_ and *C*_3_ onto the detector plane as shown in Figure 3. Let *PC*_2_ and *PC*_3_ denote these projections. The line *PC*_1_ is the projection of trajectory *C*_1_. Supp *f*(*x*) is supposed to be inside a ball centered as the origin and with sufficiently small radius *r* < *R,* so that the projection of ROI has a circular shape, as shown by the shaded region in figure 3. The curves *PC*_2_ and *PC*_3_ split the entire ROI into three sub-ROIs: *PR_m_, m* = 1,2,3. The object point x is projected into the area *PR_m_,m* = 1,2,3. Let *x̂* denotes this projection.

If *x̂* ∈ *PR*_1_, *L*(*φ*) is the projection of the plane through *x* with normal *σ_x_*(*s, φ*). As *φ* increase, *σ_x_*(*s, φ*) *φ β_⊥_*(*s, x*) rotates in the clockwise direction on *DP*(*s*). From definition of *k_x_*(*s, φ*) with equation (14) *k* is discontinuous if *L*(*φ*) is parallel to *y'*(*s*) or is tangent to *C*_2_. In the first case, such a plane has six points of intersection with *C* = *C*_1_ ∪ *C*_2_ ∪ *C*_3_, so *k* = 1/6 on the side of the jump, where *y'*(*s*) · *σ* > 0 and *k* = −1/6 on the other side. The filtering line is parallel to *y'*(*s*), which is denoted as *L*_1_(see the dot-dashed lines in Figure 3). The corresponding weighting function of filtering line *L*_1_ is *w_x_*(*s, φ*_1_) = 1/3. In the second case, the number of intersection changes form four to six. So the filtering line is tangent to *C*_2_, which is denoted as *L*_2_ (see the dot-dashed lines in Figure 3). For the signum function in Equation (14) is unchagened, the corresponding weighting function of filtering line *L*_2_ is *w_x_*(*s, φ*_2_) = 1/12. If *x̂* ∈ *PR*_2_, *k* is discontinuous only *L*(*φ*) is parallel to *y'*(*s*). So the filtering line *L*_1_ is parallel to *y*(*s*). The corresponding weight function is *w_x_*(*s, φ*_1_) = 1/3. If *x̂* ∈ *PR*_3_, *k* is discontinuous if *L*(*φ*) is parallel to *y'*(*s*) or is tangent to *C*_3_. In the first case, such a plane has six points of intersection with trajectory, so *k* = 1/6 on the side of the jump, where *y'*(*s*) · *e* > 0 and *k* = −1/6 on the other side. The filter line is parallel to *y'*(*s*), which is denoted as *L*_1_ (as shown in Figure 3).The corresponding weighting function of filtering line *L*_1_ is *w_x_*(*s, φ*_1_) = 1/3. In the second case, the number of intersection changes form four to six. So the filtering line is tangent to *C*_3_, which is denoted as *L*_3_(see the dot-dashed lines in Figure 3). The corresponding weighting function of filtering line *L*_3_ is *w_x_*(*s, φ*_3_) = 1/12. Figure 3 summarizes the above information.

## 5. Conclusions

An exact shift invariant filtered back-projection (FBP) reconstruction algorithm for a cone-beam vertex trajectory consisting of three-orthogonal to each other circular was derived from Tuy's inversion formula. There are several major modifications to Tuy's formula. The first modification is not using the Fourier transform with the projection function to deduce the specific inversion formula, but instead using the inversion Radon transform. Second, we started with a Tuy-like inversion scheme. Weighting the redundant data and rewriting the weighted summation into an integral along the source trajectory resulted in a shift-invariant FBP reconstruction formula. Last, the “nontangential” condition in Tuy's original data sufficiency conditions is relaxed and the nonended condition is further relaxed. In implementing of the inversion formula for a cone-beam vertex trajectory consisting of three-orthogonal to each other circular, the concrete forms of the filtering lines and according weighting function are the most important and difficult segments. In this paper, we obtained above two segments base on analysis about the geometric properties of projections with the three-orthogonal circular trajectory and the radon planes which pass through the reconstruction point.

## References

[1] Katsevich A. (2002). Theoretically exact filtered backprojection-type inversion algorithm for spiral CT. SIAM J. Appl. Math..

[2] Zou Y., Pan X.C. (2004). Image reconstruction on PI-lines by use of filtered backprojection in helical cone-beam CT. Phys. Med. Biol..

[3] Tuy H.K. (1983). An inverse formula for cone-beam reconstruction. J. Appl. Math..

[4] Zeng G.L., Gullberg G.T. (1992). A cone-beam tomography algorithm for orthogonal circle-and-line orbit. Phys. Med. Biol..

[5] Zeng G.L., Gullberg G.T. (1994). Implementation of Tuy's cone-beam inversion formula. Phys. Med. Biol..

[6] Ning R., Tang X., Conover D., Yu R. (2003). Flat panel detector-based cone beam computed tomography with a circle-plus-two-arcs data acquisition orbit: preliminary phantom study. Med. Phys..

[7] Wang X.H., Ning R. (1992). A cone-beam reconstruction algorithm for circle-plus-arc data-acquisition geometry. IEEE Trans. Med. Imag..

[8] Finch D.V. (1985). Cone beam reconstruction with sources on a curve. SIAM J. Appl. Math..

[9] Chen Z.K., Ning R. (2006). Volume fusion for two-circular-orbit cone-beam tomography. Appl. Opt..

[10] Noo F., Clack R., White T.A., Roney T.J. (1998). The dual-ellipse cross vertex path for exact reconstruction of long objects in cone-beam tomography. Phys. Med. Biol..

[11] Feldkamp L.A., Davis L.C., Kress J.W. (1984). Practical cone beam algorithm. J. Opt. Soc. Am..

[12] Delia S., Ivan B., Nicolas P. (2008). Studies on circular isocentric cone-beam trajectories for 3D image reconstructions using FDK algorithm. Comput. Med. Imag. Grap..

[13] Katsevich A. (2003). A general scheme for constructing inversion algorithms for cone beam CT Internat. J. Math. Math. Sci..

[14] Katsevich A. (2004). Image reconstruction for the circle and line trajectory. Phys. Med. Biol..

[15] Zamyatin A.A., Katsevich A., Chiang B.S. (2008). Exact image reconstruction for a circle and line trajectory with a gantry tilt. Phys. Med. Biol..

[16] Katsevich A. (2005). Image reconstruction for the circle-and-arc trajectory. Phys. Med. Biol..

[17] Katsevich A. (2007). Image reconstruction for a general circle-plus trajectory Inverse Problems. Inverse Probl..

[18] Katsevich A. (2004). On two version of a 3PI algorithms for helical CT. Med. Phys..

[19] Katsevich A. (2006). 3PI algorithms for helical computer tomography. Adv. Appl. Math..

[20] Grangeat P. (1991). Mathematical framework of cone beam 3D reconstruction via the first derivative of the Radon transform. In Mathematical Methods in Tomography. Lect. Notes Math..

